# Supplementary material for: Oligonucleotide-Recognizing Topoisomerase Inhibitors (OTIs): Precision Gene Editors for Neurodegenerative Diseases?
Source: Int J Mol Sci. 2022 Sep 29;23(19):11541. doi: 10.3390/ijms231911541 (PMC9570105; doi:10.3390/ijms231911541)
Supplement: Supplementary file 1 [file ijms-23-11541-s001.zip › ijms-1864290-supplementary from proof revised ref.pdf]

Supplementary material for:
'Oligonucleotide-recognizing topoisomerase inhibitors (OTIs): precision gene editors for neurodegenerative diseases?'

Ben D. Bax [1,*], Dmitry Sutormin[2], Neil Q. McDonald [3,4], Glenn A. Burley [5], and Tatyana Shelkovnikova[6]

[1] Medicines Discovery Institute, Cardiff University, Cardiff, CF10 3AT, UK.
[2] Skolkovo Institute of Science and Technology, Moscow, 121205, Russia.
[3] Signalling and Structural Biology Laboratory, The Francis Crick Institute, London NW1 1AT, UK.
[4] Institute of Structural and Molecular Biology, Department of Biological Sciences, Birkbeck College, London, WC1E 7HX, UK
[5] Department of Pure and Applied Chemistry & the Strathclyde Center for Molecular Bioscience, University of Strathclyde, Glasgow G1 1XL, UK.
[6] SITraN, Department of Neuroscience, University of Sheffield, Sheffield, S10 2HQ, UK.

Supplementary discussion: DNA topoisomerases, anti-bacterial drugs and potential developments of anti-cancer and anti-bacterial OTIs

Supplementary Table S1. Bacterial topoisomerases, DNA-cleavage stabilizing drugs and compounds in the clinic.

Supplementary Figure S1. Type IA topoisomerase structure.

Supplementary Figure S2. Human Top1: structure, function and inhibition.

Supplementary Figure S3. Modelling of a pyrrole-imidazole-polyamide (PIP) – camptothecin OTI

Supplementary Figure S4. Could T-segment binding OTIs be made to efficiently edit DNA?

Supplementary Figure S5. Could three Top1s simultaneously bind to a DNA duplex encoding amino acids 30-45 of human SOD1?

Supplementary Discussion:

DNA topoisomerases, anti-bacterial drugs and potential developments of anti-cancer and anti-bacterial OTIs

Most bacteria have four topoisomerases, two type IA (topo I and topo III), and two type IIA (DNA gyrase and topoisomerase IV) (see supplementary Table 1). By convention even numbered topoisomerases are type II, whereas odd numbered are type I. The two bacterial type IIA topoisomerases are important drug targets.

To relax positive and negative DNA supercoils and decatenate DNA molecules, type IIA topoisomerases introduce a transient four base-pair staggered double-stranded break into DNA by forming two 5' phosphotyrosine bonds (Figure 1c). The catalytic tyrosine, from a WHD domain, cleaves the DNA when it interacts with a metal binding TOPRIM domain. The bacterial type IIA topoisomerases (DNA gyrase and topoisomerase IV - supplementary Table 1) have similar structures and mechanisms to the human type IIA topoisomerases (Table 1). However, bacterial type IIA topoisomerases function as A_2B_2 heterotetramers, while eukaryotic enzymes are dimers corresponding to fusions of bacterial B and A subunits (Figure 3). Among type IIA topoisomerases, DNA gyrase has a unique ability to introduce negative supercoils into relaxed or positively supercoiled DNA due to C-terminal DNA wrapping domain [114]. This unique C-terminal domain allows DNA gyrase to use an alternative non-standard mechanism for modifying topology [115].

The quinolone/fluoroquinolone class of antibacterial drugs are one of the most successful classes of antibiotics [116] and despite a black box warning in the USA [117] new members continue to be approved [118]. Two new classes of bacterial type IIA targeting agents are currently in Phase III clinical trials (supplementary Table 1). Gepotidacin is an NBTI (non DNA-cleavage pocket binding on the twofold axis inhibitor - originally named as novel bacterial topoisomerase inhibitors) which binds midway between the two 'four base-pair staggered DNA-cleavage sites' [119-121]. Zoliflodacin is a derivative of QPT-1 [122], which binds in the two DNA-cleavage sites [63]. However, zoliflodacin interacts with GyrB residues rather than the GyrA residues recognised by fluoroquinolones [63, 123]. Dual targeting of both DNA gyrase and topoisomerase IV is important for limiting the spread of resistance. DNA gyrase/Topo IV targeting drugs, such as fluoroquinolones [124], are often broad spectrum antibiotics that can cause dysbiosis in patients.

Stabilizers of topoisomerase DNA-cleavage complexes [125] are widely used in clinical practice as anticancer drugs (e.g., camptothecins, etoposide, mitoxantrone, and doxorubicin - Table 1) and as antibacterial drugs (e.g., moxifloxacin, ciprofloxacin - supplementary Table 1) [126]. Oligonucleotide-recognizing topoisomerase inhibitors (OTIs) are bifunctional molecules, which consists of a topoisomerase inhibitor covalently linked to an oligonucleotide (or an oligonucleotide-recognizing moiety). This combines the advantages of sequence selectivity conferred by the oligonucleotide (or oligonucleotide-recognizing moiety), with the DNA-cleaving stabilizing activity of a topoisomerase inhibitor. Etoposide is used as an anti-cancer compound, and has some antibacterial activity [63]. Two types of etoposide based OTIs have been made to date [34, 43], but no antibacterial activity has yet been reported for an OTI. All OTIs made to date are derived from anti-cancer compounds (see Table 1).

Advances in DNA sequencing technologies means it is possible to sequence the genetic lesions that cause cancers [127], however, genomic instability and genomic heterogeneity is a significant issue in cancer cells [104]. Thus while it might be tempting to try to 'cut-up' the malign DNA that causes cancers and develop a human CRISPR/Cas type of immune system, what happens in human cells will depend on the DNA repair pathways [1, 128] present in the cells and such a strategy might be hazardous. Specific targeting of genes in cells with OTIs has been

reported [33, 129]. One potential use of OTIs in cancer chemotherapy would be to target drug efflux pumps such as P-glycoprotein [130] with Top1-OTIs. Safety and delivery of OTIs to cells in the patient will remain key issues. Similary targeting antimicrobial resistance (AMR) genes [131] with OTIs which only cleave DNA with bacterial topoisomerases is an attractive idea. However, safety and delivery will again be key issues. For this reason OTI development against DNA lesions in the CNS may be a good initial strategy to pursue.

Supplementary Table S1. Bacterial topoisomerases, DNA-cleavage stabilizing drugs and compounds in the clinic.

**Topoisomerase Type Polarity Mechanism**	**Gene Name**	**Protein name**	Drug (class) approx. US Approval date. (comments)	Compounds in the clinic. Phase/NTCnumber
IA<br>5'-PY Strand passage	TOPA<br>TOPB	TopoI<br>TopoIII	None yet (being sort)	None
IB<br>3'-PY Rotation	Usually none	Usually absent		
IIA<br>5'-PY Strand passage<br>ATPase	GYRA<br>GYRB<br><br>PARC<br>PARE	DNA gyrase (an A_2B_2 tetramer)<br>GyrA<br>GyrB<br><br>Topoisomerase IV (a C_2E_2 tetramer)<br>ParC<br>ParE	Quinolone/Fluoroquinolones In use since the 1960s. (Dual targetting of DNA gyrase and topoisomerase IV lowers the risk of resistance).<br>Current members include:<br>Ciprofloxacin (1987)<br>Levofloxacin (1996)<br>Moxifloxacin (1999)<br>Delafloxacin (2017)	gepotidacin (Phase III) urinary tract infection (NCT04020341)<br>Gonorrhea (NCT04010539)<br><br>zoliflodacin (Phase III) (NCT03959527)
IIB<br>5'-PY Strand passage<br>ATPase		Topo VIII	A bacterial type IIB topoisomerase, called Topo VIII, is only present on mobile genetic elements. It may be involved in horizontal gene transfer of antimicrobial resistance genes [132, 133].	None
