# Supplementary material for: Oligonucleotide-Recognizing Topoisomerase Inhibitors (OTIs): Precision Gene Editors for Neurodegenerative Diseases?
Source: Int J Mol Sci. 2022 Sep 29;23(19):11541. doi: 10.3390/ijms231911541 (PMC9570105; doi:10.3390/ijms231911541)
Supplement: Supplementary file 1 [file ijms-23-11541-s001.zip › review-figS1-suppl-12Sept2022b.pdf]

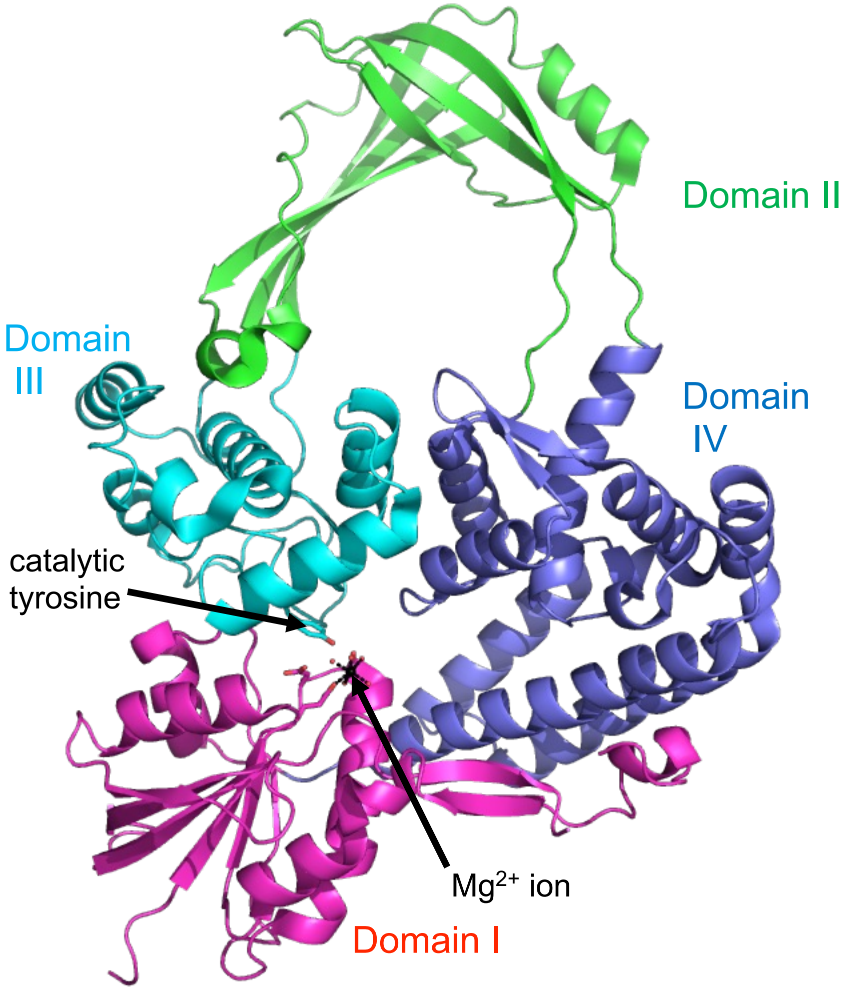


**Supplementary Figure S1. Type IA topoisomerase structure.**

A type IA topoisomerase structure. The 2.44Å crystal structure of human TOP3β (pdb code: 5gvc) has a typical 'toroidal' structure for a type IA topoisomerase. The catalytic tyrosine (from domain III) and a $Mg^{2+}$ ion (on domain I) are arrowed and indicate the active site where single strand DNA-cleavage takes place. A recent paper reports that TOP3β is required efficient replication of positive-sense RNA-viruses (Prasanth, K.R., et al., Topoisomerase III-β is required for efficient replication of positive-sense RNA viruses. Antiviral research, 2020. 182: p. 104874).
