# Supplementary material for: Oligonucleotide-Recognizing Topoisomerase Inhibitors (OTIs): Precision Gene Editors for Neurodegenerative Diseases?
Source: Int J Mol Sci. 2022 Sep 29;23(19):11541. doi: 10.3390/ijms231911541 (PMC9570105; doi:10.3390/ijms231911541)
Supplement: Supplementary file 1 [file ijms-23-11541-s001.zip › review-figS2-suppl-28July2022b.pdf]

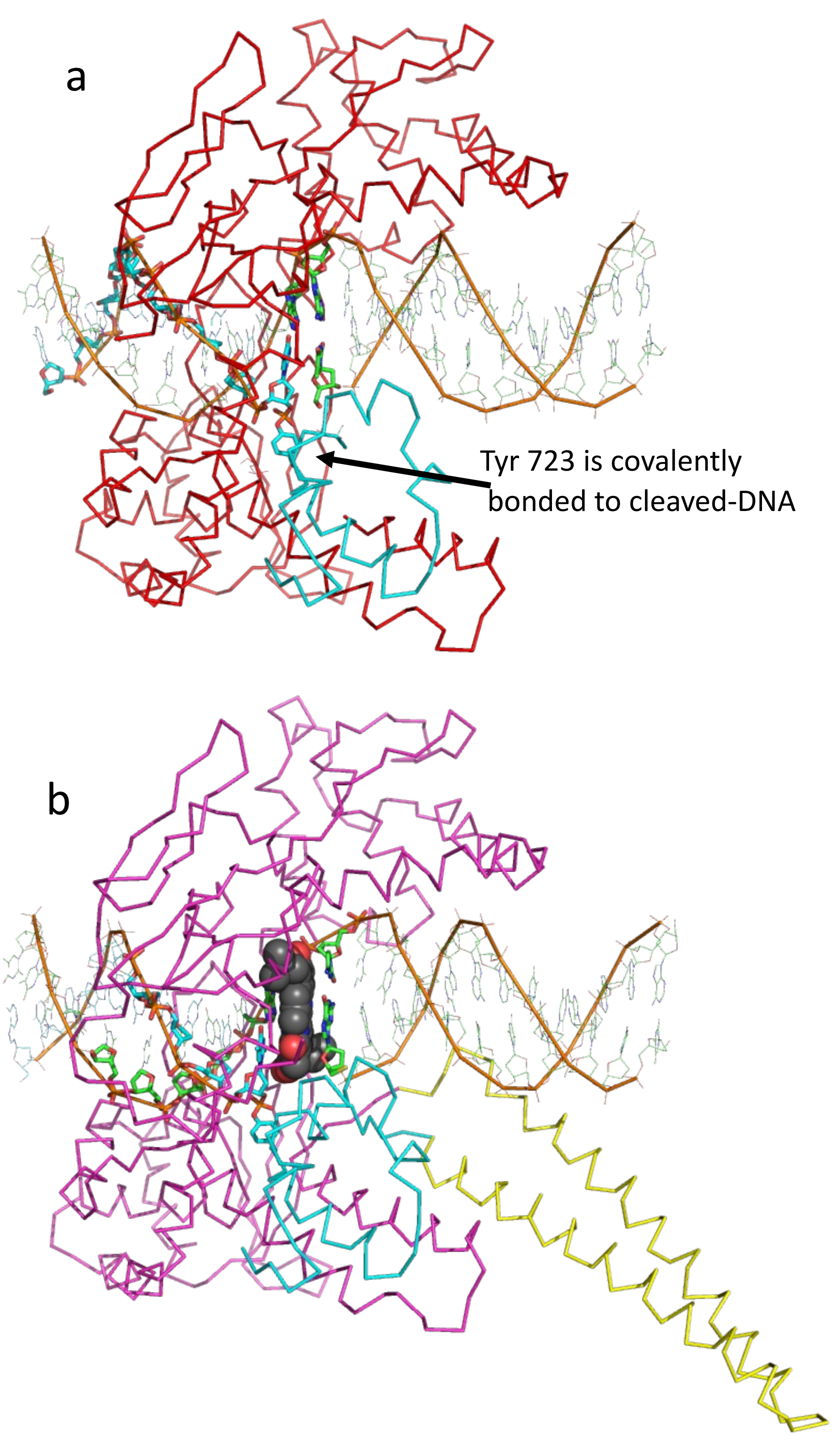

Supplementary Figure S2. Human Top1: structure, function and inhibition.

(**a**) A Top1 structure with DNA. The 2.1Å structure of a truncated form of human topoisomerase I (residues 215-635 red Cα trace, 713-765 cyan Cα trace) in a DNA-cleavage complex (pdb code: 1a31); the DNA is shown as backbone ribbon is shown together with thin ‘lines’ (green carbons) for the DNA. The tyrosine 723 (stick - arrowed) that has cleaved the DNA and is covalently linked to the DNA backbone is shown as fatter ‘sticks’, as are nucleotides on either side of the DNA-cleavage site. **(b)** An equivalent view of a 2.1Å DNA-cleavage complex of human topoisomerase I with topotecan (pdb code: 1k4t). The compound is shown in grey space-fill, between the base-pairs at the DNA-cleavage site. The linker domain of topoisomerase I (residues 636-712) is shown in yellow Cα trace. The major contacts between the protein and the DNA are with main-chain phosphates of the DNA on one-side of the DNA-cleavage site (main-chain DNA in contact region shown as sticks).
