# Supplementary material for: Oligonucleotide-Recognizing Topoisomerase Inhibitors (OTIs): Precision Gene Editors for Neurodegenerative Diseases?
Source: Int J Mol Sci. 2022 Sep 29;23(19):11541. doi: 10.3390/ijms231911541 (PMC9570105; doi:10.3390/ijms231911541)
Supplement: Supplementary file 1 [file ijms-23-11541-s001.zip › review-figS3-suppl-28July2022b.pdf]

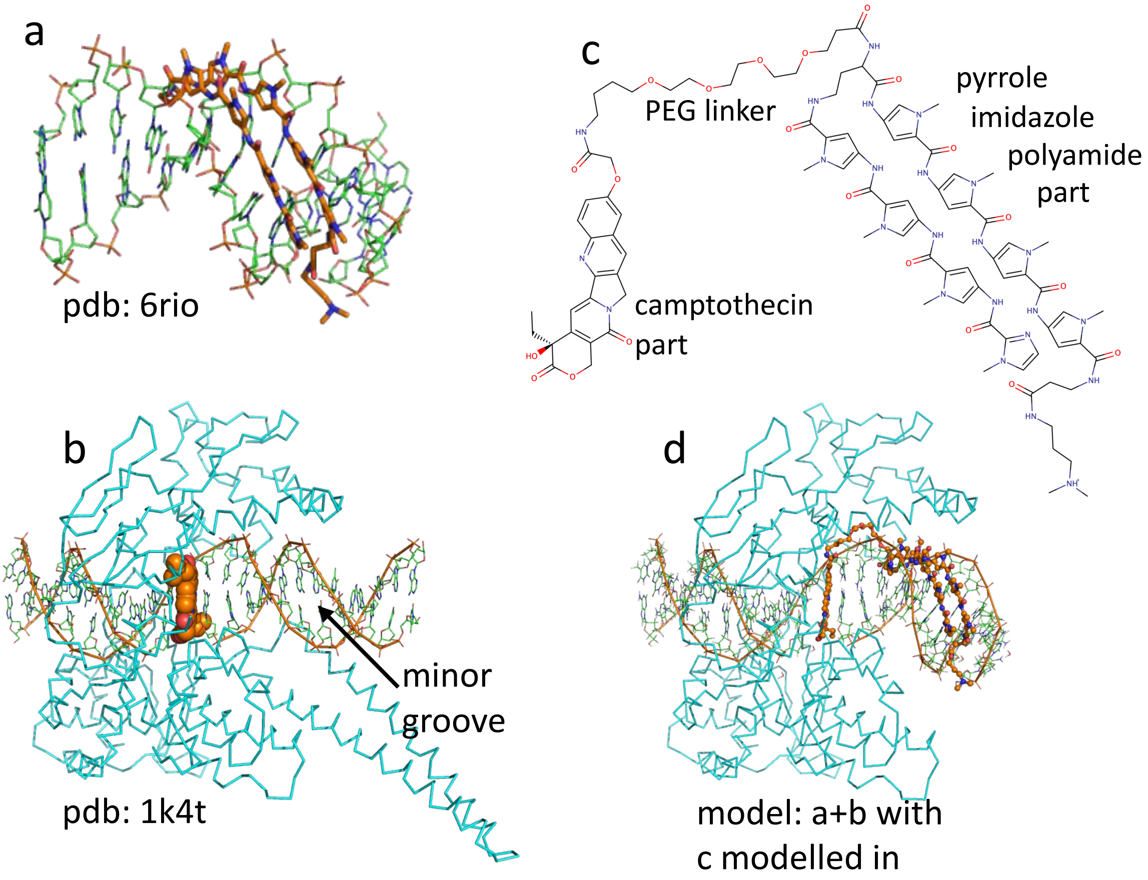

Supplementary Figure S3. Modelling of a pyrrole-imidazole-polyamide (PIP) – camptothecin OTI.

(**a**) NMR structure – first model only (pdb code: 6rio) of a PIP bound in the 'minor' groove of a target DNA duplex (**b**) A 2.1Å DNA-cleavage complex of human Top1 with topotecan (pdb code: 1k4t). The compound is shown in orange space-fill, between the base-pairs at the DNA-cleavage site (same view of same structure as in Supplementary Figure 2b). **(c)** A hairpin polyamide-camptothecin conjugate from Wang, and Dervan (2001, *Sequence-specific trapping of topoisomerase I by DNA binding polyamide–camptothecin conjugates.* J. Am. Chem. Soc., 123, 8657-8661) is shown - picture drawn with Marvin-sketch (from ChemAxon (https://www.chemaxon.com) (**d**) The DNA – hairpin polyamide from 6rio has been modelled on the DNA in 1k4t (note this increases the width of the minor groove) and the PEG linker has been modelled in (with Maestro - Schrödinger Release 2020-2: Maestro, Schrödinger, LLC, New York, NY, 2021). The linker domain (residues 636-712) has been deleted from the model (see supplementary figure 2a – for a picture of a crystal structure in which this domain has been deleted).
