# Supplementary material for: Oligonucleotide-Recognizing Topoisomerase Inhibitors (OTIs): Precision Gene Editors for Neurodegenerative Diseases?
Source: Int J Mol Sci. 2022 Sep 29;23(19):11541. doi: 10.3390/ijms231911541 (PMC9570105; doi:10.3390/ijms231911541)
Supplement: Supplementary file 1 [file ijms-23-11541-s001.zip › review-figS4-suppl-28July2022b.pdf]

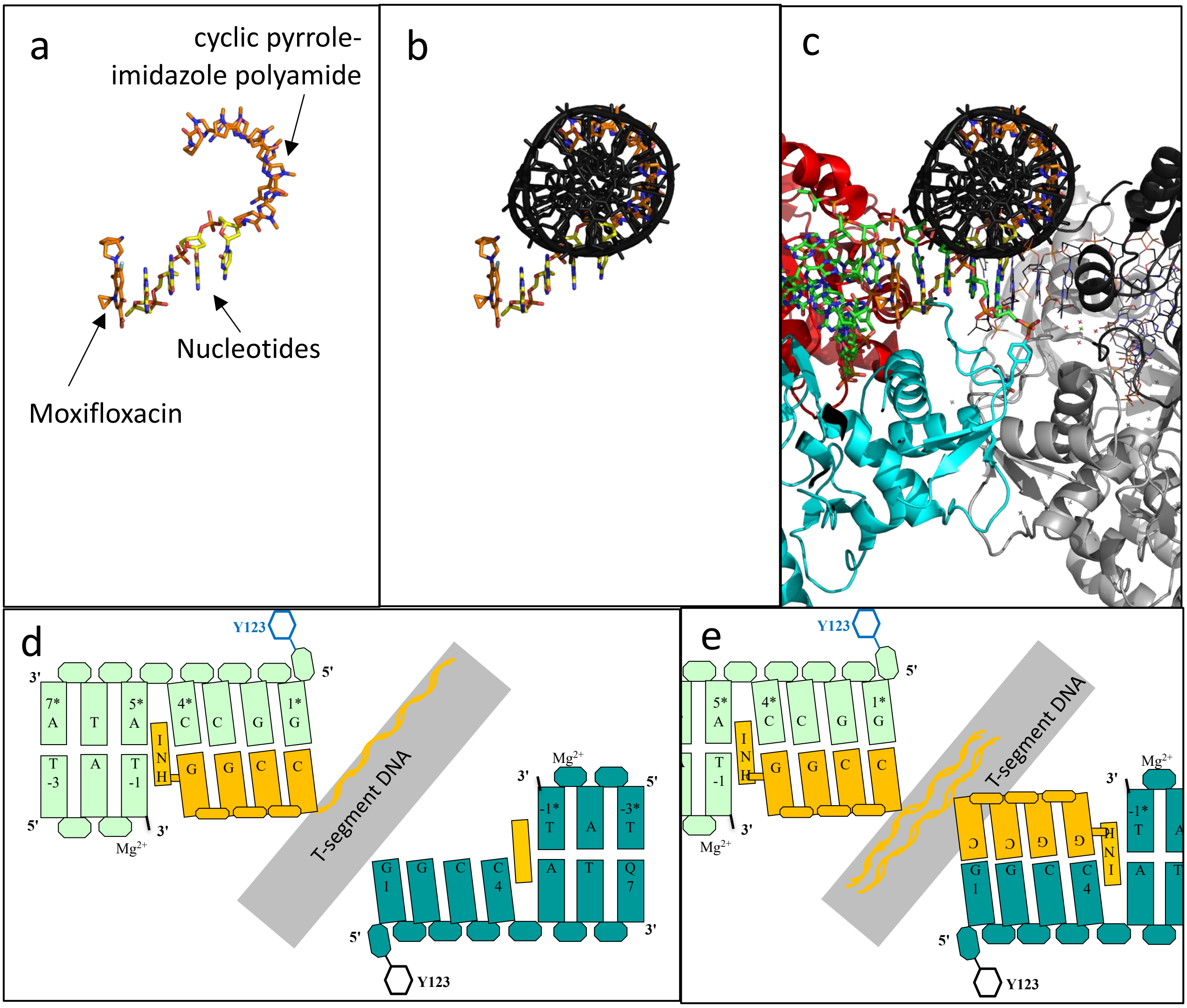

Supplementary Figure S4 Could T-segment binding OTIs be made to efficiently edit DNA?

A short OTI has been modelled with moxifloxaicin covalently coupled to a minor groove recognising pyrrole-imidazole polyamide (PIP) binding a T-segment. **(a)** Proposed molecules contain three covalently linked (covalent links not shown) components: (i) moxifloxacin (orange carbons – from pdb code: 5cdq) (ii) nucleotides (yellow carbons: 5cdq) and (iii) minor groove recognising pyrrole-imidazole polyamide (orange carbons- 6m5b) – to bind to the T-segment. (b) the cyclic polyamide is shown bound in the minor groove of a DNA segment (DNA coloured black – pdb code: 6m5b). (c) The four nucleotide and compound are shown interacting with cleaved DNA (pdb code: 5cdq). The cleaved DNA is covalently attached to tyrosine 123. (d) schematic of c – T-segment DNA is shown in grey. (e) schematic with two OTIs attached to a single pyrrole-imidazole polyamide.
