# Supplementary material for: Oligonucleotide-Recognizing Topoisomerase Inhibitors (OTIs): Precision Gene Editors for Neurodegenerative Diseases?
Source: Int J Mol Sci. 2022 Sep 29;23(19):11541. doi: 10.3390/ijms231911541 (PMC9570105; doi:10.3390/ijms231911541)
Supplement: Supplementary file 1 [file ijms-23-11541-s001.zip › review-figS5-suppl-28July2022b.pdf]

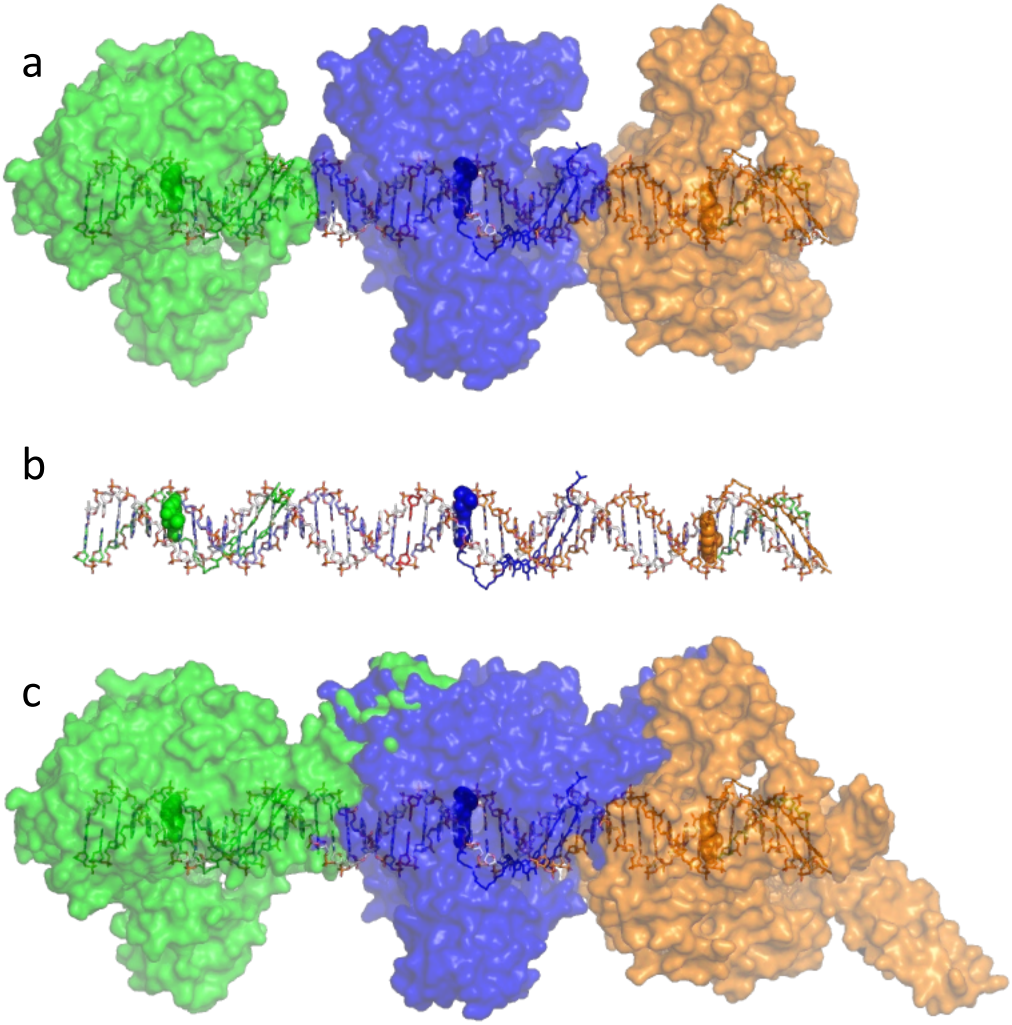

Supplementary Figure S5. Could three human Top1s simultaneoulsy bind to a DNA duplex encoding amino acids 30-45 of human SOD1?

a. A rough model of three Top1s on a model of (BDNA - from http://www.scfbio-iitd.res.in/software/drugdesign/bdna.jsp) encoding amino acids 30-45 of human SOD1. The figure is as in figure 7, but with three protein subunits shown in space-fill at 40% transparency.. The modelling suggests it may be possible to fit three Top1 subunits simultaneously, but experiiments would be needed to prove this possibilty. Models were made by superposing three nucleotides adjacent to the DNA-cleavage site from the model of the **pyrrole-imidazole-polyamide (PIP) – camptothecin OTI** (see supplementary figure 3) onto three base-pairs adjacent to DNA-cleavage sites in the B-DNA model. **b** The B-DNA model is shown in stick with carbons coloured on the cleaved DNA-strand to show the three DNA-cleavage sites. Three PIP-OTIs are shown in green, blue and orange, with the PIP and linker moieties shown in stick and the camptothecin moieties in space-fill. Note that the DNA will be distorted by the binding of the PIP-OTIs, shown with the pyrrole imidazole polyamide moiety in the minor groove of the B-DNA and the space-filling camptothecin moiety at the three DNA-cleavage sites. **c** The conformationally flexible linker domain (residues 636-712) has been included by superposing pdb code 1k4t – on models shown in panel a. Note a large clash between the green linker domain and the blue subunit in this model. However, no distortions have been introduced into the B-DNA – which will distort on binding the PIP and the Top1.
